# Severe Maternal Morbidity and Mental Health Hospitalizations or Emergency Department Visits

**DOI:** 10.1001/jamanetworkopen.2024.7983

**Published:** 2024-04-23

**Authors:** Asia Blackman, Ugochinyere V. Ukah, Robert W. Platt, Xiangfei Meng, Gabriel D. Shapiro, Isabelle Malhamé, Joel G. Ray, Sarka Lisonkova, Darine El-Chaâr, Nathalie Auger, Natalie Dayan

**Affiliations:** 1 Department of Epidemiology, Biostatistics and Occupational Health, McGill University, Montreal, Québec, Canada; 2 Pregnancy and Child Research Center, HealthPartners Institute, Minneapolis, Minnesota; 3 Centre for Outcomes Research and Evaluation, Research Institute of the McGill University Health Centre, Montreal, Québec, Canada; 4 Department of Medicine, McGill University Health Centre, Montreal, Québec, Canada; 5 Department of Psychiatry, McGill University, Montreal, Québec, Canada; 6 Douglas Research Centre, Montreal, Québec, Canada; 7 Department of Medicine, University of Toronto, Toronto, Ontario, Canada; 8 Department of Obstetrics and Gynaecology, University of British Columbia, Vancouver, British Columbia, Canada; 9 Department of Obstetrics and Gynaecology, University of Ottawa, Ottawa, Ontario, Canada; 10 Institut national de santé publique du Québec, Montréal, Québec, Canada

## Abstract

**Importance:**

Severe maternal morbidity (SMM) can have long-term health consequences for the affected mother. The association between SMM and future maternal mental health conditions has not been well studied.

**Objective:**

To assess the association between SMM in the first recorded birth and the risk of hospitalization or emergency department (ED) visits for a mental health condition over a 13-year period.

**Design, Setting, and Participants:**

This population-based retrospective cohort study used data from postpartum individuals aged 18 to 55 years with a first hospital delivery between 2008 and 2021 in 11 provinces and territories in Canada, except Québec. Data were analyzed from January to June 2023.

**Exposure:**

SMM, defined as a composite of conditions, such as septic shock, severe preeclampsia or eclampsia, severe hemorrhage with intervention, or other complications, occurring after 20 weeks’ gestation and up to 42 days after a first delivery.

**Main Outcomes and Measures:**

The main outcome was a hospitalization or ED visit for a mental health condition, including mood and anxiety disorders, substance use, schizophrenia, and other psychotic disorder, or suicidality or self-harm event, arising at least 43 days after the first birth hospitalization. Cox regression models generated hazard ratios with 95% CIs, adjusted for baseline maternal comorbidities, maternal age at delivery, income quintile, type of residence, hospital type, and delivery year.

**Results:**

Of 2 026 594 individuals with a first hospital delivery, 1 579 392 individuals (mean [SD] age, 30.0 [5.4] years) had complete ED and hospital records and were included in analyses; among these, 35 825 individuals (2.3%) had SMM. Compared with individuals without SMM, those with SMM were older (mean [SD] age, 29.9 [5.4] years vs 30.7 [6.0] years), were more likely to deliver in a teaching tertiary care hospital (40.8% vs 51.1%), and to have preexisting conditions (eg, ≥2 conditions: 1.2% vs 5.3%), gestational diabetes (8.2% vs 11.7%), stillbirth (0.5% vs 1.6%), preterm birth (7.7% vs 25.0%), or cesarean delivery (31.0% vs 54.3%). After a median (IQR) duration of 2.6 (1.3-6.4) years, 1287 (96.1 per 10 000) individuals with SMM had a mental health hospitalization or ED visit, compared with 41 779 (73.2 per 10 000) individuals without SMM (adjusted hazard ratio, 1.26 [95% CI, 1.19-1.34]).

**Conclusions and Relevance:**

In this cohort study of postpartum individuals with and without SMM in pregnancy and delivery, there was an increased risk of mental health hospitalizations or ED visits up to 13 years after a delivery complicated by SMM. Enhanced surveillance and provision of postpartum mental health resources may be especially important after SMM.

## Key Points

**Question:**

Is severe maternal morbidity (SMM) associated with long-term mental health–related hospitalizations or emergency department (ED) visits after delivery?

**Findings:**

In this cohort study among 1 579 392 individuals with hospital births in Canada, 35 825 individuals had SMM within pregnancy or up to 42 days post partum, and 1 543 567 individuals did not. SMM was associated with a 1.3-fold increased rate of hospitalization or ED visit for a mental health condition up to 13 years post partum.

**Meaning:**

These findings suggest that SMM was associated with adverse mental health conditions beyond the conventional postpartum period.

## Teaser

This cohort study assesses the association between severe maternal morbidity after a first delivery and risk of hospitalization or emergency department (ED) visits for a mental health condition over a 13-year period.

## Introduction

Severe maternal morbidity (SMM) includes life-threatening conditions occurring in pregnancy or soon after delivery, such as severe hemorrhage, severe preeclampsia or eclampsia, and septic shock.^1,2,3,4^ Beyond the known increased risks of short-term mortality and prolonged hospital stay in individuals with SMM compared with unaffected individuals,^5,6^ those who survive SMM are more likely to develop chronic health conditions, including cardiovascular disease,^6,7^ long-term impaired functional ability, and chronic pain.^8,9^ The trauma of SMM and its consequences could adversely affect psychological health.^10^ Given that up to 36% of mortality in the first year post partum is due to suicide,^11^ it is critical to assess the mental health burden after SMM.

Studies of associations between various adverse pregnancy events and postpartum mental health outcomes have principally examined specific conditions, like preeclampsia, or have relatively short follow-up periods.^12,13^ Furthermore, very few studies have been completed within Canada,^12,13,14,15^ which has a multiethnic population with universal antenatal health coverage yet variable access to postpartum mental health services. Knowledge of the short- and long-term risks of serious mental health conditions after SMM and its subtypes could inform the need for enhanced postpartum supportive resources. We therefore performed a population-based cohort study assessing mental health hospitalizations and emergency department (ED) visits over a 13-year period, comparing individuals who experienced SMM with unaffected postpartum individuals in Canada.

## Methods

This cohort study was approved by the research ethics board of the McGill University Health Centre (MUHC). Since the study used secondary aggregate data, the need for individual informed consent was waived by MUHC. We followed the Strengthening the Reporting of Observational Studies in Epidemiology (STROBE) reporting guideline.

### Study Design and Data Sources

We conducted a cohort study of individuals with a first recorded hospital delivery between April 1, 2008, and March 31, 2021. Data were extracted from the Canadian Institute of Health Information (CIHI) Discharge Abstract Database (DAD), including administrative, clinical, and demographic information on all hospital deliveries within Canada, excluding Québec, as this province does not submit hospitalization data to CIHI. The DAD accounts for approximately 98% of deliveries in Canada outside Québec.^16,17^ While most mental health hospitalizations are also typically recorded in the DAD, in some Ontario facilities, these events are reported through the Ontario Mental Health Reporting System, a data source that was unavailable at the time of this study. The accuracy of DAD records has been validated against medical records, demonstrating high specificity and sensitivity for most maternal conditions and high specificity and low to moderate sensitivity for most mental health conditions.^18,19^

Using unique patient identifiers, the CIHI DAD was linked to the National Ambulatory Care Reporting System (NACRS) dataset, which includes patient data from EDs.^16,20^ Because coverage of ED data is variable across provinces and fiscal year,^20,21^ ED outcomes were assessed among individuals with available NACRS data (eFigure 1 in Supplement 1). Up to 25 diagnostic and 20 procedural codes per visit were captured using *International Statistical Classification of Diseases and Related Health Problems, Tenth Revision, Canadian version (ICD-10-CA) *and the Canadian Classification of Health Interventions (CCI) respectively. Diagnostic and procedural codes were used to characterize the sample and define study exposures and outcomes.^16,22^

### Study Population

Individuals aged 18 to 55 years with a first recorded liveborn or stillborn delivery with pregnancy lasting between 20 and 43 weeks’ gestation were included.^23^ Individuals who delivered after 43 weeks’ gestation and those younger than 18 or older than 55 years at delivery were excluded to optimize the accuracy of obstetric codes; individuals younger than 18 or older than 55 years were also excluded to account for unique mental health experience among pregnancies at extremes of age.^24,25^ As the interest was in new-onset mental health visits related to SMM, the primary analysis excluded individuals with a previous mental health hospitalization or ED visit within 2 years before the index birth, an approach that has been used previously.^12,26,27^ Duplicate delivery records, delivery records with missing identifiers, and individuals with ectopic pregnancy or miscarriage were also excluded. Therapeutic abortions and out-of-hospital deliveries were not available in the data source, but out-of-hospital deliveries account for less than 2% of deliveries in Canada.^17^

### Study Exposure

Exposure to SMM was captured between 20 weeks’ gestation and 42 days after delivery hospital discharge in the first recorded hospital birth; individuals without SMM were considered unexposed. SMM was defined using the validated Canadian Perinatal Surveillance System definition,^1^ which included 1 or more of the following diagnoses: severe preeclampsia or eclampsia; severe hemorrhage; cardiac complications (cardiomyopathy, cardiac arrest, resuscitation, myocardial infarction, pulmonary edema, and heart failure); complications of anesthesia; surgical complications; cerebrovascular accidents; acute kidney failure; embolism, shock, or disseminated intravascular coagulation; severe sepsis; uterine rupture; acute fatty liver or liver failure; cerebral edema; and coma. SMM also included critical illness interventions: urgent hysterectomy, dialysis, assisted ventilation, and intensive care unit (ICU) admission (eTable 1 in Supplement 1); SMM indicators are not mutually exclusive. Chronic HIV and prevalent hypertensive heart disease were excluded from our definition of SMM, as our interest was in the study of acute conditions with onset during pregnancy and the postpartum period.^1,28^ Acute psychosis was excluded from the SMM definition as this was a component of the primary outcome.

### Study Outcomes

The primary outcome was a composite of mental health hospitalizations or ED visits occurring 43 days or more after the index birth hospitalization, defined by a primary or secondary coded diagnosis in the DAD for mood or anxiety disorder, substance-related or addictive disorder, schizophrenia spectrum or other psychotic disorder, or suicidality or self-harm event^29^ (eTable 2 in Supplement 1). Secondary outcomes were individual components of the primary outcome.

### Covariates

Covariates selected a priori as potential confounders in multivariable models were captured within 2 years prior to index birth hospitalization, and guided by a directed acyclic graph (eFigure 2 in Supplement 1). These included maternal age at delivery, income quintile, delivery year, province or territory of delivery, maternal comorbid conditions (preexisting hypertension; diabetes; chronic kidney disease; chronic liver disease; cardiovascular condition; sickle cell disease; HIV; autoimmune syndrome, such as systemic lupus erythematosus; asthma; obesity; or smoking), urban or rural residential status, and hospital type (teaching tertiary care hospital vs community hospital).^30^

### Statistical Analysis

Baseline characteristics for the cohort were described, stratified according to the presence or absence of SMM in first recorded birth, using means and SDs or medians and IQRs for continuous data and frequencies and percentages for categorical data. A graph was generated displaying the temporal trend in frequency and rates per 1000 deliveries of SMM in all births by fiscal year.

Incidence rates were calculated per 10 000 person-years with 95% CIs for the composite outcome and individual components in individuals with SMM and unaffected individuals. Univariable and multivariable Cox proportional hazards models were used to calculate crude hazard ratios (HRs) and adjusted HRs (aHRs) and 95% CIs, estimating the association between SMM and a mental health hospitalization or ED visit.

In secondary analyses, the association between SMM and each component of the primary outcome was assessed. Also, the risks of outcomes according to common individual SMM diagnoses were examined. Finally, associations of SMM with hospitalizations and with ED visits were assessed separately, as individuals requiring hospitalization may differ in important ways from individuals with an ED visit with subsequent discharge to the community.^31^ In all models, follow-up was censored on death or end of study period (March 2021). Follow-up was also censored on subsequent pregnancy in the primary analysis to address misclassification bias arising from individuals who experienced SMM in subsequent pregnancies but not the first.

A complete-case analysis was conducted after we determined that 2.4% of the records in the dataset had missing values and deleting them would be unlikely to meaningfully impact estimates.^32^ Baseline characteristics were compared among those with and without missing data to assess the validity of our findings. Log(−log[survival]) by log(time) plots were generated to test the proportional hazards assumption.

We performed 7 sensitivity analyses. First, we assessed hospitalizations with an *ICD-10-CA* code for a mental health condition in any of the 25 DAD fields to capture visits with as opposed to for a mental health condition.^33^ Second, we reincluded individuals with preexisting mental health conditions identified prior to the index pregnancy in the cohort and stratified models according to the presence or absence of a previous mental health hospitalization or ED visit to assess for exacerbated disease. Third, we excluded Ontario births from the cohort, because mental health hospitalizations in this province are reported largely through the Ontario Mental Health Reporting System.^21,33^ Fourth, we ran a model in which there was no censoring on a subsequent pregnancy, as some individuals at high risk for the outcome of interest may be less likely to conceive or have longer interpregnancy intervals. Fifth, we excluded stillbirths and preterm births from the cohort, as these events have been associated with postpartum mental illness. Sixth, we ran a model in which SMM was limited to events occurring at or before delivery and follow-up started the day after hospital discharge. Finally, we ran models for 3 separate follow-up periods: up to 1 year, 1 to 5 years, and more than 5 years, to assess how proximity to delivery might modify the association between SMM and mental health hospitalization or ED visit.

*P* values were 2-sided, and statistical significance was set at *P* = .01. Data were analyzed using SAS software version 9.4 (SAS Institute). Data were analyzed from January to June 2023.

## Results

### Baseline Characteristics of Study Sample

**Figure 1.**
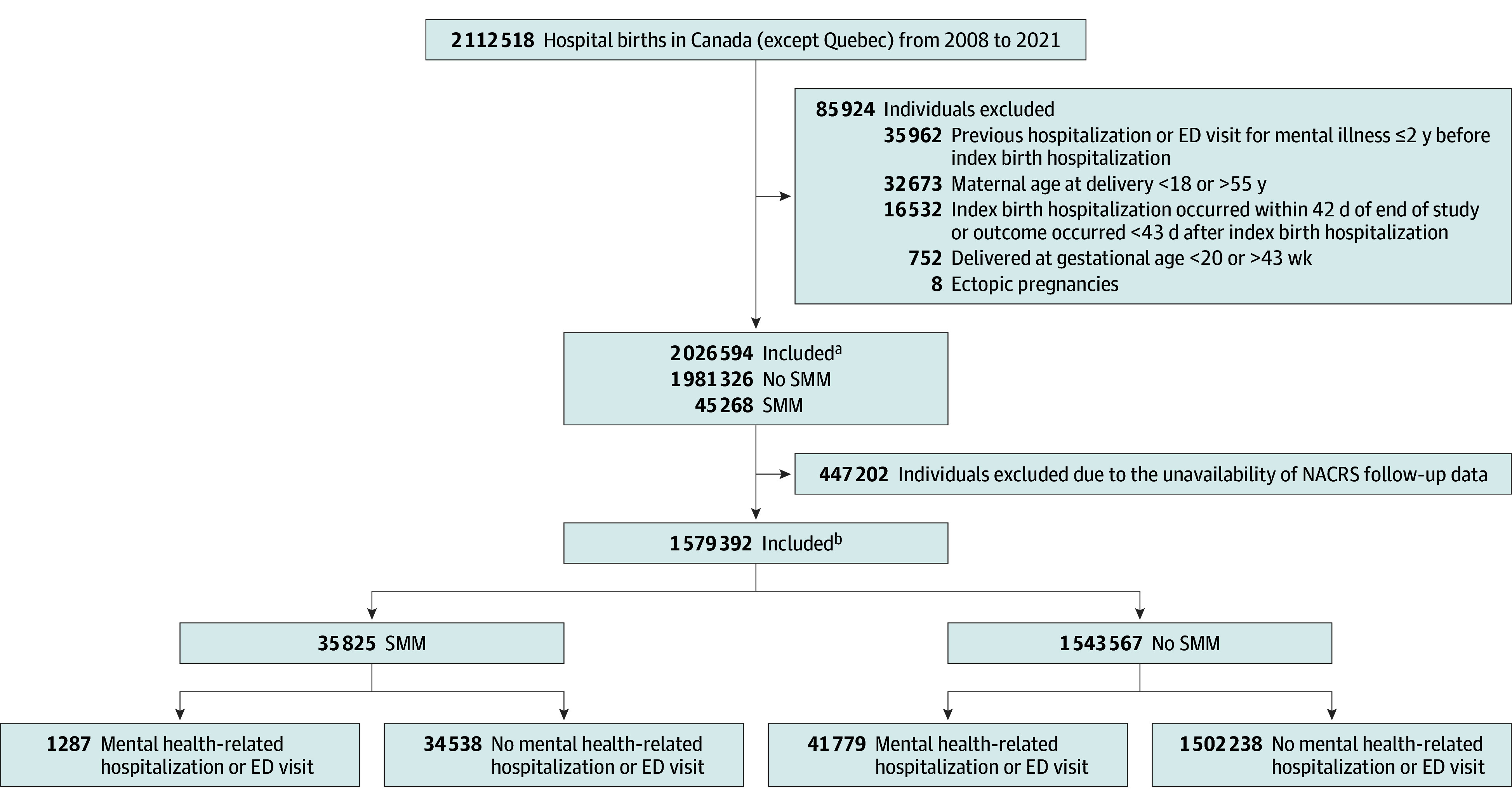
Study Flowchart NACRS indicates National Ambulatory Care Reporting System; SMM, severe maternal morbidity. ^a^This cohort was used to calculate outcomes of mental health hospitalization alone. ^b^This cohort was used to calculate outcomes that included emergency department (ED) visits, ie, mental health hospitalization and/or mental health ED visit.

We identified 2 026 594 individuals (mean [SD] age, 29.8 [5.4] years) with a first hospital delivery (45 268 individuals [2.3%] with SMM). After restricting the cohort to individuals whose index delivery occurred in a province and year with available NACRS data, the analytic cohort included 1 579 392 individuals, of whom 35 825 (2.3%) had SMM (Figure 1). Compared with individuals without SMM, those with SMM were older (mean [SD] age, 29.9 [5.4] years vs 30.7 [6.0] years), were more likely to deliver in a teaching tertiary care hospital (40.8% vs 51.1%), and to have preexisting conditions (eg, ≥2 conditions: 1.2% vs 5.3%), gestational diabetes (8.2% vs 11.7%), stillbirth (0.5% vs 1.6%), preterm birth (7.7% vs 25.0%), or cesarean delivery (31.0% vs 54.3%) (Table 1).The overall SMM rate increased from 15.5 per 1000 deliveries in 2006 to 2007 to 22.2 per 1000 deliveries in 2020 to 2021 (eFigure 3 in Supplement 1).

**Table 1.** Baseline Characteristics of Study Cohort at First Recorded Hospital Delivery, Stratified According to the Absence or Presence of Severe Maternal Morbidity

Characteristic	Individuals, No. (%) (N = 1 579 392)		Standardized difference
	No SMM (n = 1 543 567)	SMM (n = 35 825)	
Maternal age at delivery, y			
Mean (SD)	29.9 (5.4)	30.7 (6.0)	NA
18-24	250 882 (16.3)	5621 (15.7)	−0.02
25-29	465 906 (30.2)	9215 (25.7)	−0.10
30-34	520 051 (33.7)	11 493 (32.1)	−0.03
35-39	246 750 (16)	6948 (19.4)	0.09
40-44	55 723 (3.6)	2209 (6.2)	0.12
≥45	4255 (0.3)	339 (0.9)	0.08
Income quintile			
Lowest	353 546 (22.9)	9038 (25.2)	0.05
Second	316 698 (20.5)	7289 (20.3)	0.00
Third	314 151 (20.4)	7065 (19.7)	−0.02
Fourth	294 826 (19.1)	6599 (18.4)	−0.02
Highest quintile	229 072 (14.8)	5028 (14)	−0.02
Missing	35 274 (2.3)	806 (2.2)	−0.01
Delivery year			
2008-2009	115 082 (7.5)	2021 (5.6)	−0.08
2009-2010	98 629 (6.4)	1756 (4.9)	−0.07
2010-2011	87 345 (5.7)	1613 (4.5)	−0.05
2011-2012	83 853 (5.4)	1719 (4.8)	−0.03
2012-2013	113 608 (7.4)	2800 (7.8)	0.02
2013-2014	117 359 (7.6)	2842 (7.9)	0.01
2014-2015	140 139 (9.1)	3253 (9.1)	0.00
2015-2016	137 703 (8.9)	3457 (9.6)	0.02
2016-2017	136 206 (8.8)	3280 (9.2)	0.01
2017-2018	134 031 (8.7)	3406 (9.5)	0.03
2018-2019	131 963 (8.5)	3386 (9.5)	0.03
2019-2020	134 380 (8.7)	3312 (9.2)	0.02
2020-2021	113 269 (7.3)	2980 (8.3)	0.04
Province			
Alberta	221 857 (14.4)	6152 (17.2)	0.08
British Colombia	156 489 (10.1)	3920 (10.9)	0.03
Manitoba	64 181 (4.2)	1640 (4.6)	0.02
Nova Scotia	60551 (3.9)	1339 (3.7)	−0.01
Ontario	971 530 (62.9)	21 028 (58.7)	−0.09
Prince Edward Island	9102 (0.6)	194 (0.5)	−0.01
Saskatchewan	50 974 (3.3)	1320 (3.7)	0.02
Northern Territories	8883 (0.6)	232 (0.6)	0.00
Urban or rural residence			
Urban	1 340 153 (86.8)	30 450 (85)	−0.05
Rural or remote	168 849 (10.9)	4588 (12.8)	0.06
Missing	34 565 (2.2)	787 (2.2)	0.00
Hospital type			
Teaching tertiary hospital	629 329 (40.8)	18 301 (51.1)	0.21
Community	903 527 (58.5)	17 264 (48.2)	−0.21
Missing	10 611 (0.7)	260 (0.7)	0.00
Comorbidity^a^			
0	1 330 804 (86.2)	26 327 (73.5)	−0.32
1	194 715 (12.6)	7587 (21.2)	0.23
≥2	18 048 (1.2)	1911 (5.3)	0.23
Nonsevere hypertensive disorders of pregnancy			
Yes	89 730 (5.8)	3545 (9.9)	0.15
No	1 453 837 (94.2)	32 280 (90.1)	−0.15
Gestational diabetes			
Yes	127 201 (8.2)	4179 (11.7)	0.12
No	1 416 366 (91.8)	31 646 (88.3)	−0.12
Gestational age at delivery, wk			
Median (IQR)	39(38-40)	38(37-40)	NA
≤22	3409 (0.2)	227 (0.6)	0.06
22-27	9571 (0.6)	1234 (3.4)	0.20
28-32	13 821 (0.9)	2009 (5.6)	0.27
33-36	91 540 (5.9)	5479 (15.3)	0.31
≥37	1 424 744 (92.3)	26 863 (75.0)	−0.48
Missing	482 (<0.1)	13 (<0.1)	NA
Preterm birth^b^			
Yes	118 341 (7.7)	8949 (25.0)	0.48
No	1 424 744 (92.3)	26 863 (75.0)	−0.48
Missing	482 (<0.1)	13 (<0.1)	0.00
Stillbirth^c^			
Yes	7573 (0.5)	559 (1.6)	0.11
No	1 535 994 (99.5)	35 266 (98.4)	−0.11
Delivery mode			
Cesarean	477 751 (31.0)	19 459 (54.3)	0.48
Vaginal	851 997 (55.2)	11 513 (32.1)	−0.48
Obstetric delivery not otherwise specified	213 819 (13.9)	4853 (13.5)	−0.01

Abbreviations: NA, not applicable; SMM, severe maternal morbidity.

^a^ Comorbidity includes preexisting hypertension, diabetes, chronic kidney disease, chronic liver disease, cardiovascular condition, sickle cell disease, HIV, autoimmune syndrome (eg, systemic lupus erythematosus), asthma, obesity, or smoking.

^b^ Preterm birth is defined as gestational age at delivery less than 37 weeks.

^c^ Stillbirth is defined as pregnancy loss at gestational age more than 20 weeks.

### Incidence Rates of Mental Health–Related Hospitalizations or ED Visits

A total of 43 066 individuals with recent childbirth (73.2 per 10 000 person-years) were hospitalized or visited the ED for a mental health condition (68.7 per 10 000 ED visits and 13.0 per 10 000 hospitalizations). Frequencies of hospital or ED visits for individual mental health diagnoses were 34 997 individuals (59.2 per 10 000 person-years) with mood and anxiety disorders, 10 240 individuals (17.1 per 10 000 person-years) with substance abuse and other related disorders, 2888 individuals (4.8 per 10 000 person-years) with suicidality or self-harm, and 2501 individuals (4.1 per 10 000 person-years) with schizophrenia spectrum or other psychotic disorders. Rates of mental health hospitalization or ED visit decreased with increasing age and income quintile and were highest among individuals living in rural or remote areas, individuals who had 2 or more comorbid medical conditions, and individuals who experienced preterm birth, stillbirth, or vaginal delivery at the index birth (Table 2).

**Table 2.** Frequency and Incidence Rates of Mental Health Hospitalizations or ED Visits According to Characteristics of First Recorded Hospital Delivery

Characteristic	No.		Incidence rate per 10 000 person-years (95% CI)
	Mental health–related hospitalizations or ED visits	Person-years	
Total cohort	43 066	5 879 735	73.2 (72.6-73.9)
Maternal age at delivery, y			
18-24	14 922	871 259	171.3 (168.5-174.0)
25-29	11 113	1 543 777	72.0 (70.6-73.3)
30-34	9903	1 929 888	51.3 (50.3-52.3)
35-39	5564	1 188 251	46.8 (45.6-48.1)
40-44	1462	322 437	45.3 (43.0-47.7)
≥45	102	24 122	42.3 (34.1-50.5)
Income quintile			
Lowest	13 299	1 435 654	92.6 (91.1-94.2)
Second	9382	1 223 606	76.7 (75.1-78.2)
Third	8078	1 172 466	68.9 (67.4-70.4)
Fourth	6761	1 070 181	63.2 (61.7-64.7)
Highest	4699	852 590	55.1 (53.5-56.7)
Missing	847	125 238	67.6
Urban or rural residence			
Urban	34 834	5 180 595	67.2 (66.5-68.0)
Rural or Remote	7421	576 117	128.8 (125.9-131.7)
Missing	811	123 023	65.9
Hospital type			
Teaching tertiary care hospital	14 658	2 290 466	64.0 (63.0-65.0)
Community hospital	27 792	3 539 042	78.5 (77.6-79.5)
Missing	616	50 228	122.6
Comorbidity^a^			
0	35 264	5 065 994	69.6 (68.9-70.3)
1	6722	738 654	91.0 (88.8-93.2)
≥2	1080	75 088	143.8 (135.3-152.4)
Nonsevere hypertensive disorders of pregnancy			
Yes	2731	345 853	789.0 (76.0-81.9)
No	40 335	5 533 882	72.9 (72.2-73.6)
Gestational diabetes			
Yes	3064	476 944	64.24 (61.97-66.52)
No	40 002	5 402 791	74.04 (73.32-74.77)
Gestational age at delivery, wk			
≤23	133	9893	134.4 (111.6-157.3)
23-28	384	42 355	90.7 (81.6-99.7)
28-33	591	64 899	91.1 (83.7-98.4)
33-36	3451	383 662	90.0 (87.0-93.0)
≥36	38 471	5 376 772	71.6 (70.8-72.3)
Missing	36	2155	167.1
Preterm birth^b^			
Yes	4559	500 809	91.0 (88.4-93.7)
No	38 471	5 376 772	71.6 (70.8-72.3)
Missing	36	2154	167.1
Stillbirth^c^			
Yes	245	20 979	116.8 (102.2-131.4)
No	42 821	5 858 756	73.1 (72.4-73.8)
Delivery mode			
Cesarean	13 438	1 916 645	70.1 (68.9-71.3)
Vaginal	24 750	3 228 415	76.7 (75.7-77.6)
Obstetric delivery NOS	4878	734 675	66.4 (64.5-68.3)

Abbreviations: ED, emergency department; NOS, not otherwise specified.

^a^ Comorbidity includes preexisting hypertension, diabetes mellitus, chronic kidney disease, chronic liver disease, cardiovascular condition, sickle cell disease, HIV, autoimmune syndrome such as systemic lupus erythematosus, asthma, obesity or smoking.

^b^ Preterm birth is defined as gestational age at delivery less than 37 weeks.

^c^ Stillbirth is defined as pregnancy loss at gestational age less than 20 weeks.

### Association Between SMM and Mental Health–Related Hospitalization or ED Visit

**Figure 2.**
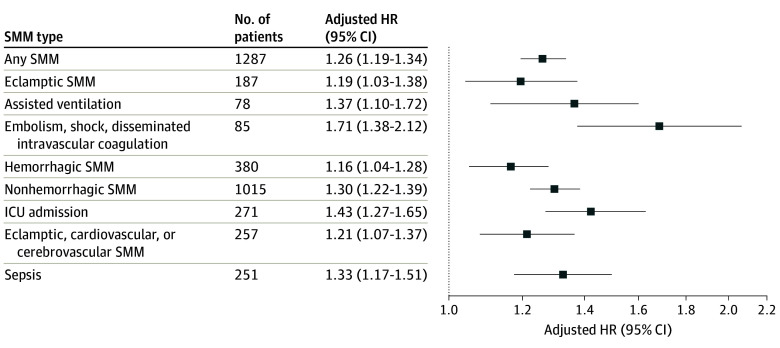
Forest Plot of Adjusted Hazard Ratios for the Association of Individual Severe Maternal Morbidity (SMM) Indicators and Mental Health Hospitalization or Emergency Department (ED) Visit Adjusted hazard ratios (HRs) compare individuals with SMM with the reference group of individuals without SMM. ICU indicates intensive care unit.

Overall, 1287 (96.1 per 10 000) individuals with SMM and 41 779 (72.7 per 10 000) unaffected individuals had a mental health hospitalization or ED visit (HR, 1.31 [95% CI, 1.24-1.39]; aHR, 1.26 [95% CI, 1.19-1.34]) (Table 3). The median (IQR) time to event was 2.8 (1.3-6.5) years for individuals with SMM-affected deliveries and 2.6 (1.3-6.4) years for individuals with deliveries without SMM. The relative increased risk after SMM was seen for all components of the mental health outcome except for schizophrenia spectrum and other psychotic disorder. The greatest risk of hospitalization or ED visit was observed for suicidality and self-harm (aHR, 1.54 [95% CI, 1.26-1.88]) (Table 3). The risk of a mental health hospitalization or ED visit was highest if the SMM subtype was embolism, shock, and disseminated intravascular coagulation (aHR. 1.71 [95% CI, 1.38-2.12]). Procedural indicators of SMM, such as use of assisted ventilation and maternal ICU admission, were similarly associated with mental health outcomes (Figure 2).

**Table 3.** Severe Maternal Morbidity and Associated Rate and Risk of Mental Health–Related Hospitalization or ED Visits, 2008 to 2021, Overall and for Specific Mental Health Diagnoses (N = 1 579 392)

SMM	Mental health hospitalization or ED visit^a^					Mental health hospitalization^b^					Mental health ED visit^a^				
	No.	PYs	Incidence rate per 10 000 PYs	Crude HR (95% CI)	Adjusted HR (95% CI)^c^	No.	PYs	Incidence rate per 10 000 PYs	Crude HR (95% CI)	Adjusted HR (95% CI)^c^	No.	PYs	Incidence rate per 10 000 PYs	Crude HR (95% CI)	Adjusted HR (95% CI)^c^
**Any mental health condition**																
Overall	43 066	5 879 735	73.2	NA	NA	11 176	85,97 956	13.0	NA	NA	40 432	5 887 822	68.7	NA	NA
SMM	1287	133 969	96.1	1.31 (1.24-1.39)	1.26 (1.19-1.34)	388	194 634	19.9	1.57 (1.35-1.81)	1.47 (1.33-1.63)	1193	134 267	88.9	1.29 (1.22-1.37)	1.25 (1.18-1.33)
No SMM	41 779	5 745 766	72.7	1 [Reference]	1 [Reference]	10 788	8 402 962	12.8	1 [Reference]	1 [Reference]	39 239	5 753 556	68.2	1 [Reference]	1 [Reference]
**Mood or anxiety disorder**																
Overall	34 997	5 907 143	59.2	NA	NA	6575	8 614 661	7.6	NA	NA	33 260	5 912 667	56.3	NA	NA
SMM	1016	134 861	75.3	1.28 (1.20-1.36)	1.23 (1.16-1.31)	230	195 179	11.8	1.56 (1.37-1.78)	1.49 (1.31-1.70)	959	135 034	71.0	1.27 (1.19-1.35)	1.23 (1.15-1.31)
No SMM	33 981	5 772 282	58.9	1 [Reference]	1 [Reference]	6345	8 419 482	7.54	1 [Reference]	1 [Reference]	32 301	5 777 633	55.9	1 [Reference]	1 [Reference]
**Schizophrenia spectrum and other psychotic disorders**																
Overall	2501	6 028 454	4.2	NA	NA	1295	8 637 258	1.5	NA	NA	2142	6 029 524	3.6	NA	NA
SMM	70	138 352	5.1	1.23 (0.97-1.57)	1.18 (0.93-1.50)	47	195 921	2.4	1.63 (1.21-2.17)	1.53 (1.13-2.06)	51	138 425	3.7	1.04 (0.79-1.37)	1.02 (0.77-1.34)
No SMM	2431	5 890 102	4.1	1 [Reference]	1 [Reference]	1248	8 441 338	1.5	1 [Reference]	1 [Reference]	2091	5 891 098	3.6	1 [Reference]	1 [Reference]
**Substance abuse and other related disorders**																
Overall	10 240	6 004 512	17.1	NA	NA	3774	8 628 700	4.4	NA	NA	9306	6 007 100	15.5	NA	NA
SMM	313	137 636	22.7	1.30 (1.16-1.46)	1.22 (1.09-1.38)	132	195 631	6.8	1.56 (1.31-1.86)	1.46 (1.22-1.74)	283	137 734	20.6	1.33 (1.19-1.50)	1.23 (1.09-1.39)
No SMM	9927	5 866 877	16.9	1 [Reference]	1 [Reference]	3642	8 433 069	4.3	1 [Reference]	1 [Reference]	9023	5 869 366	15.4	1 [Reference]	1 [Reference]
**Suicidality or self-harm**																
Overall	2888	6 027 271	4.8	NA	NA	1272	8 637 163	1.5	NA	NA	2559	6 028 407	4.2	NA	NA
SMM	106	138 258	7.7	1.62 (1.33-1.97)	1.54 (1.26-1.88)	48	195 937	2.5	1.65 (1.23-2.23)	1.57 (1.16-2.12)	91	138 301	6.6	1.57 (1.27-1.93)	1.50 (1.21-1.86)
No SMM	2782	5 889 013	4.7	1 [Reference]	1 [Reference]	1224	8 441 226	1.5	1 [Reference]	1 [Reference]	2468	5 890 106	4.2	1 [Reference]	1 [Reference]

Abbreviations: ED, emergency department; HR, hazard ratio; NA, not applicable; PY, person-year; SMM, severe maternal morbidity.

^a^ Alberta contributed ED data for follow-up from 2010 to 2021, British Columbia contributed ED data for follow-up from 2008 to 2010 and 2011 to 2021, Manitoba contributed ED data for follow- up from 2009 to 2021, New Brunswick contributed no ED data for follow-up, Newfoundland and Labrador contributed no ED data for follow-up, Nova Scotia contributed ED data for follow-up from 2008 to 2021, Ontario contributed ED data for follow-up from 2008 to 2021, Saskatchewan contributed ED data for follow-up from 2010 to 2021, and the Northern Territories contributed ED data for follow-up from 2008 to 2021. Data were available for 1 579 392 women overall, including 35 825 with SMM and 1 543 567 without SMM.

^b^ Data were available for 2 026 594 women overall, including 45 268 with SMM and 1 981 326 without SMM.

^c^ HRs adjusted for maternal age at delivery, income quintile, comorbidity, delivery year and urban or rural residential status.

While ED visits were more frequent than hospitalizations for all mental health conditions, the relative risks for ED visit and hospitalization after SMM were similarly elevated (Table 3). Similar increased relative risks for mental health hospitalizations and mental health ED visits were seen across all provinces examined (eTable 3 in Supplement 1).

### Sensitivity Analyses

The relative risks of hospitalization using the broader definition for a mental health condition (ie, a mental health diagnostic code in any of the 25 diagnostic fields) were similar to that in the primary analysis (eTable 4 in Supplement 1). Absolute rates of mental health visits were substantially higher in individuals with vs without previous mental illness within the last 2 years, but relative risks were not appreciably different from the primary analysis (eTable 5 in Supplement 1). Similarly, the removal of births in Ontario did not significantly impact our findings (eFigure 4 in Supplement 1) nor did ignoring subsequent pregnancy (eTable 6 in Supplement 1), excluding preterm births and stillbirths from the cohort (eTable 7 in Supplement 1), or starting follow-up the day after hospital discharge (eTable 8 in Supplement 1). Individuals with SMM had the highest relative risk of hospitalization or ED visit for a mental health condition in the first year post partum (aHR, 1.38 [95% CI, 1.24-1.53]). Individuals with more than 1 year and less than 5 years of follow-up had increased risk of hospitalization or ED visit for a mental health condition (aHR, 1.23 [95% CI 1.14-1.34]), as did those with more than 5 years of follow-up (aHR, 1.21 [95% CI, 1.07-1.37]) (eTable 9 in Supplement 1).

### Missing Data Assessment

Overall, 2.43% of records in the dataset had missing data for at least 1 study variable. Baseline characteristics comparing 1 541 105 individuals with complete data with 38 287 individuals with incomplete data were overall similar, except that individuals with missing data were younger at the time of the index birth, were more often from the lowest income quintile and rural or remote areas, and delivered in the years 2019, 2020, or 2021 (eTable 10 in Supplement 1).

## Discussion

In this population-based Canadian cohort study, individuals who experienced SMM had increased risk of mental health hospitalization or ED visit up to 13 years after delivery compared with those who did not experience SMM. This increase in risk was consistent across provinces and for both hospitalizations and ED visits. The risk of a mental health hospitalization or ED visit was highest during the first postpartum year and among individuals who were treated in a maternal ICU during pregnancy and those with an embolism, shock, or disseminated intravascular coagulation. Individuals with SMM had the highest risk of hospitalization or ED visit for suicidality and self-harm.

These results corroborate and expand on findings from previous work conducted in the US and Sweden.^12,13^ A 2019 study by Lewkowitz et al^12^ analyzed 1 229 835 pregnant individuals in Florida and found that SMM was associated with a 74% higher odds of hospital admission for depression, anxiety, and psychosis in the first year post partum. Unlike our study, Lewkowitz et al^12^ did not find an association between SMM and suicidality, likely due to differences in sample size. The Swedish study by Wall-Wieler et al^13^ included 25 674 deliveries and found that SMM was associated with higher odds of inpatient psychiatric treatment for mood disorders, neuroses, and behavioral disorders also in the first postpartum year. Estimates from the analysis by Wall-Wieler et al^13^ (adjusted odds ratio, 1.22 [95% CI, 1.03-1.45) are similar to those in this study, although Wall-Wieler et al did not adjust for maternal comorbidity.

SMM comprises heterogeneous indicators of traumatic, near-fatal pregnancy-related events that can be disruptive to mental health through various pathways, including disruptions to the hypothalamic-pituitary-axis (HPA) and the hypothalamic-pituitary-gonadal (HPG) axis following SMM, which may lead to clinically apparent depression, anxiety, and psychosis.^34,35,36,37^ Maternal ICU admission often co-occurs with SMM, resulting in separation of parent and neonate and subsequent maternal distress.^38,39,40^ Also, individuals with SMM are less likely to experience factors associated with protection against mental illness, like exclusive breastfeeding, mother-infant bonding, and adequate sleep.^8,9,41,42,43,44,45,46^

This study has several strengths including the large sample size, which facilitated the analysis of a rare exposure and a rare outcome. The population-based nature of the study increases the generalizability of our findings, while the relatively long follow-up period and the use of robust, validated definitions enhanced their internal validity.

### Limitations

This study has some limitations, primarily related to the observational design using administrative health data. Due to lack of data on ambulatory clinic visits and prescriptions, we could not capture individuals with less severe health conditions, such as some mood and anxiety disorders and other comorbid conditions that are commonly managed in outpatient settings. This could have led to both misclassification of outcomes and unmeasured confounding by comorbidities and previous mental illness. In an effort to capture the whole of pregnancy morbidity, our cohort included both stillbirths and live births, as well as term and preterm births. We acknowledge that mothers who experienced stillbirth or extreme prematurity would be more at risk for mental health issues. In sensitivity analyses restricted to term liveborn deliveries, the relative risks of SMM on mental health visits were unchanged. Therefore, our findings suggest that SMM, with or without stillbirth and preterm birth, was associated with future mental health visits in the mother. We acknowledge that our principal results apply mostly to individuals with intermediate or high risk; however, findings of a sensitivity analysis demonstrating similar relative risks of the outcome due to SMM among individuals with preexisting mental illness suggest that SMM was a risk factor associated with poor mental health regardless of baseline predisposition.

Results may not be generalizable to all births. We acknowledge that individuals with the most severe SMM subtypes may be less likely to conceive again and more likely to have psychological complications; however, our results were consistent in models in which we did not censor on subsequent pregnancy. Operational definitions used to define SMM in CIHI DAD have been validated several times over the past 15 years.^47,48,49^ Any recent changes to diagnostic definitions may not be captured, but there have been no major changes to SMM diagnoses since 2016. Despite few missing data, we acknowledge the possibility that we may have misestimated the true associations due to exclusion of individuals with incomplete data. Additionally, this study does not capture data on completed suicide and thus may underestimate the severity of mental health issues after SMM.

## Conclusions

In this cohort study of postpartum individuals with and without SMM in pregnancy, SMM was associated with hospitalization or ED visit for mental health conditions several years after obstetric delivery. These findings suggest that individuals who experience severe pregnancy complications may benefit from additional mental health screening.

## Notes

Supplement 1. **eFigure 1.** Data Availability by Province or Territory Across the Study Period **eFigure 2.** Directed Acyclic Graph Demonstrating the Relationships Between Study Variables **eFigure 3.** Frequency of Severe Maternal Morbidity and Rate per Thousand Births Among Hospital Deliveries in Canada (Excluding Quebec) During Fiscal Years 2006-07 Through 2020-21 **eFigure 4.** Forest Plot of Hazard Ratios for the Relationship Between SMM and Mental Health Hospitalization or ED Visit, by Province or Territory **eTable 1.** Exposure Definition With Accompanying *ICD-10* and CCI Codes **eTable 2.** Outcome Definition With Accompanying *ICD-10* and CCI Codes **eTable 3.** Association Between Severe Maternal Morbidity and the Risk of Mental Health Hospitalization, or Mental Health ED Visit, Separately, by Province or Territory **eTable 4.** Association Between Severe Maternal Morbidity and the Risk of Hospitalization and/or ED Visit Capturing a Mental Health Condition Recorded in Any of the 25 Diagnostic Fields During Hospitalization **eTable 5.** Association Between Severe Maternal Morbidity and the Risk of Mental Health Hospitalization or ED Visit Among Individuals With Previous Mental Illness **eTable 6.** Association Between Severe Maternal Morbidity and the Risk of Mental Health Hospitalization or ED Visit Without Censoring on Subsequent Pregnancy **eTable 7.** Association Between Severe Maternal Morbidity and the Risk of Mental Health Hospitalization or ED Visit in a Cohort Where Stillbirth and Preterm Birth Are Excluded **eTable 8.** Association Between Severe Maternal Morbidity at or Before Delivery and the Risk of Mental Health Hospitalization or ED Visit **eTable 9.** Association Between Severe Maternal Morbidity and the Risk of Mental Health-Related Hospitalization or ED Visit Within the First Year Post Partum, From 1 Year to 5 Years Post Partum, and Beyond 5 Years Post Partum **eTable 10.** Baseline Characteristics of Study Cohort at First Recorded Hospital Delivery, Stratified According to the Absence or Presence of Missing Data

Supplement 2.
Data Sharing Statement
