# Error in Author Affiliations

**DOI:** 10.1001/jamanetworkopen.2024.18594

**Published:** 2024-05-21

In the Original Investigation titled “Severe Maternal Morbidity and Mental Health Hospitalizations or Emergency Department Visits,”^1^ published April 23, 2024, there was an error in the affiliations for authors Nathalie Auger, MD, and Natalie Dayan, MD. Dr Auger’s affiliation should have appeared as Institut national de santé publique du Québec, Montréal, Québec, Canada. Dr Dayan’s affiliations should have included the Department of Medicine, McGill University Health Centre, Montreal, Québec, Canada. This article has been corrected.^1^
