# Supplementary material for: Severe Maternal Morbidity and Mental Health Hospitalizations or Emergency Department Visits
Source: JAMA Netw Open. 2024 Apr 23;7(4):e247983. doi: 10.1001/jamanetworkopen.2024.7983 (PMC11040413; doi:10.1001/jamanetworkopen.2024.7983)
Supplement: Supplement 1. — eFigure 1. Data Availability by Province or Territory Across the Study Period eFigure 2. Directed Acyclic Graph Demonstrating the Relationships Between Study Variables eFigure 3. Frequency of Severe Maternal Morbidity and Rate per Thousand Births Among Hospital Deliveries in Canada (Excluding Quebec) During Fiscal Years 2006-07 Through 2020-21 eFigure 4. Forest Plot of Hazard Ratios for the Relationship Between SMM and Mental Health Hospitalization or ED Visit, by Province or Territory eTable 1. Exposure Definition With Accompanying ICD-10 and CCI Codes eTable 2. Outcome Definition With Accompanying ICD-10 and CCI Codes eTable 3. Association Between Severe Maternal Morbidity and the Risk of Mental Health Hospitalization, or Mental Health ED Visit, Separately, by Province or Territory eTable 4. Association Between Severe Maternal Morbidity and the Risk of Hospitalization and/or ED Visit Capturing a Mental Health Condition Recorded in Any of the 25 Diagnostic Fields During Hospitalization eTable 5. Association Between Severe Maternal Morbidity and the Risk of Mental Health Hospitalization or ED Visit Among Individuals With Previous Mental Illness eTable 6. Association Between Severe Maternal Morbidity and the Risk of Mental Health Hospitalization or ED Visit Without Censoring on Subsequent Pregnancy eTable 7. Association Between Severe Maternal Morbidity and the Risk of Mental Health Hospitalization or ED Visit in a Cohort Where Stillbirth and Preterm Birth Are Excluded eTable 8. Association Between Severe Maternal Morbidity at or Before Delivery and the Risk of Mental Health Hospitalization or ED Visit eTable 9. Association Between Severe Maternal Morbidity and the Risk of Mental Health-Related Hospitalization or ED Visit Within the First Year Post Partum, From 1 Year to 5 Years Post Partum, and Beyond 5 Years Post Partum eTable 10. Baseline Characteristics of Study Cohort at First Recorded Hospital Delivery, Stratified According to the Absence or Presence of Missing [file jamanetwopen-e247983-s001.pdf]

Supplemental Online Content

Blackman A, Ukah UV, Platt RW, et al. Severe maternal morbidity and mental health hospitalizations or emergency department visits. *JAMA Netw Open*. 2024;7(4):e247983. doi:10.1001/jamanetworkopen.2024.7983

eFigure 1. Data Availability by Province or Territory Across the Study Period

eFigure 2. Directed Acyclic Graph Demonstrating the Relationships Between Study Variables

eFigure 3. Frequency of Severe Maternal Morbidity and Rate per Thousand Births Among Hospital Deliveries in Canada (Excluding Quebec) During Fiscal Years 2006-07 Through 2020-21

eFigure 4. Forest Plot of Hazard Ratios for the Relationship Between SMM and Mental Health Hospitalization or ED Visit, by Province or Territory

eTable 1. Exposure Definition With Accompanying ICD-10 and CCI Codes

eTable 2. Outcome Definition With Accompanying ICD-10 and CCI Codes

eTable 3. Association Between Severe Maternal Morbidity and the Risk of Mental Health Hospitalization, or Mental Health ED Visit, Separately, by Province or Territory

eTable 4. Association Between Severe Maternal Morbidity and the Risk of Hospitalization and/or ED Visit Capturing a Mental Health Condition Recorded in Any of the 25 Diagnostic Fields During Hospitalization

eTable 5. Association Between Severe Maternal Morbidity and the Risk of Mental Health Hospitalization or ED Visit Among Individuals With Previous Mental Illness

eTable 6. Association Between Severe Maternal Morbidity and the Risk of Mental Health Hospitalization or ED Visit Without Censoring on Subsequent Pregnancy

eTable 7. Association Between Severe Maternal Morbidity and the Risk of Mental Health Hospitalization or ED Visit in a Cohort Where Stillbirth and Preterm Birth Are Excluded

eTable 8. Association Between Severe Maternal Morbidity at or Before Delivery and the Risk of Mental Health Hospitalization or ED Visit

eTable 9. Association Between Severe Maternal Morbidity and the Risk of Mental Health-Related Hospitalization or ED Visit Within the First Year Postpartum, From 1 Year to 5 Years Postpartum, and Beyond 5 Years Postpartum

eTable 10. Baseline Characteristics of Study Cohort at First Recorded Hospital Delivery, Stratified According to the Absence or Presence of Missing Data

This supplemental material has been provided by the authors to give readers additional information about their work.

© 2024 Blackman A et al. *JAMA Network Open.*

eFigure 1. Data Availability by Province/Territory Across the Study Period

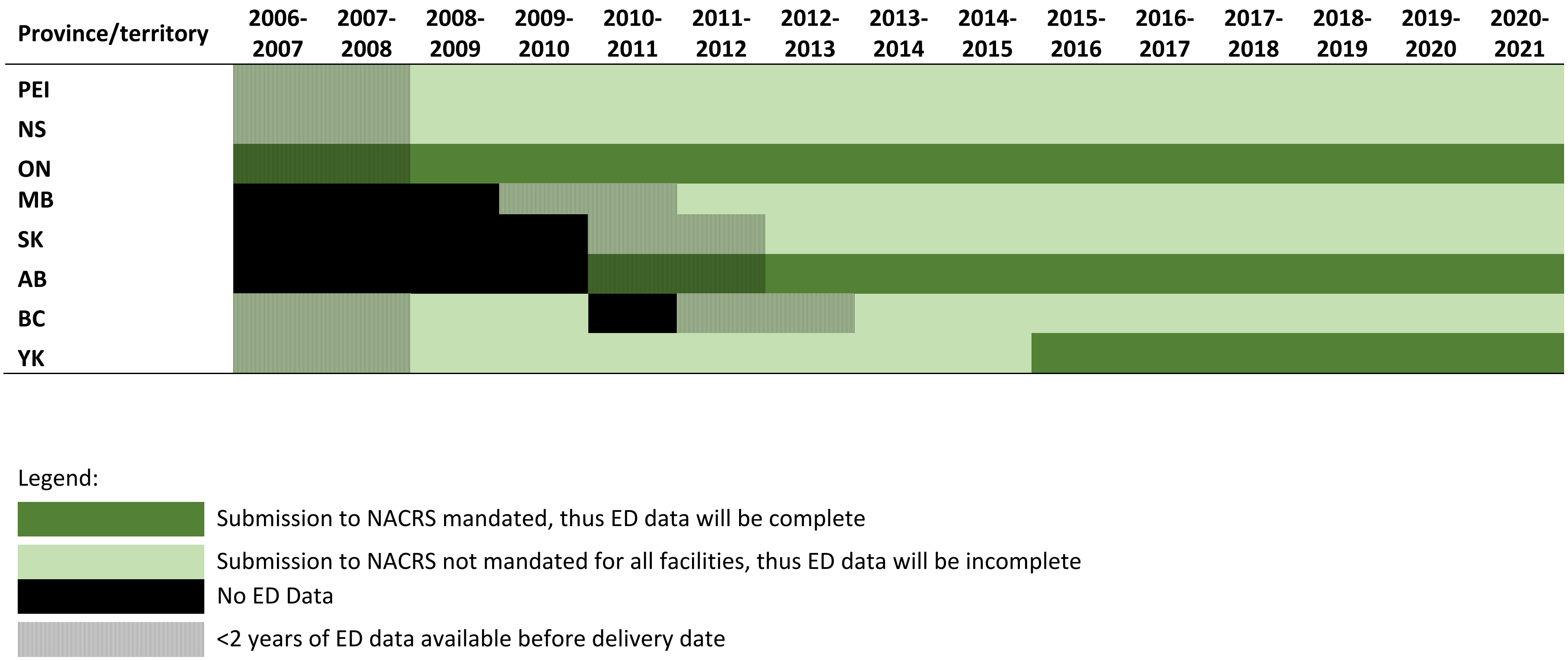

© 2024 Blackman A et al. *JAMA Network Open.*

eFigure 2. Directed Acyclic Graph Demonstrating the Relationships between Study Variables

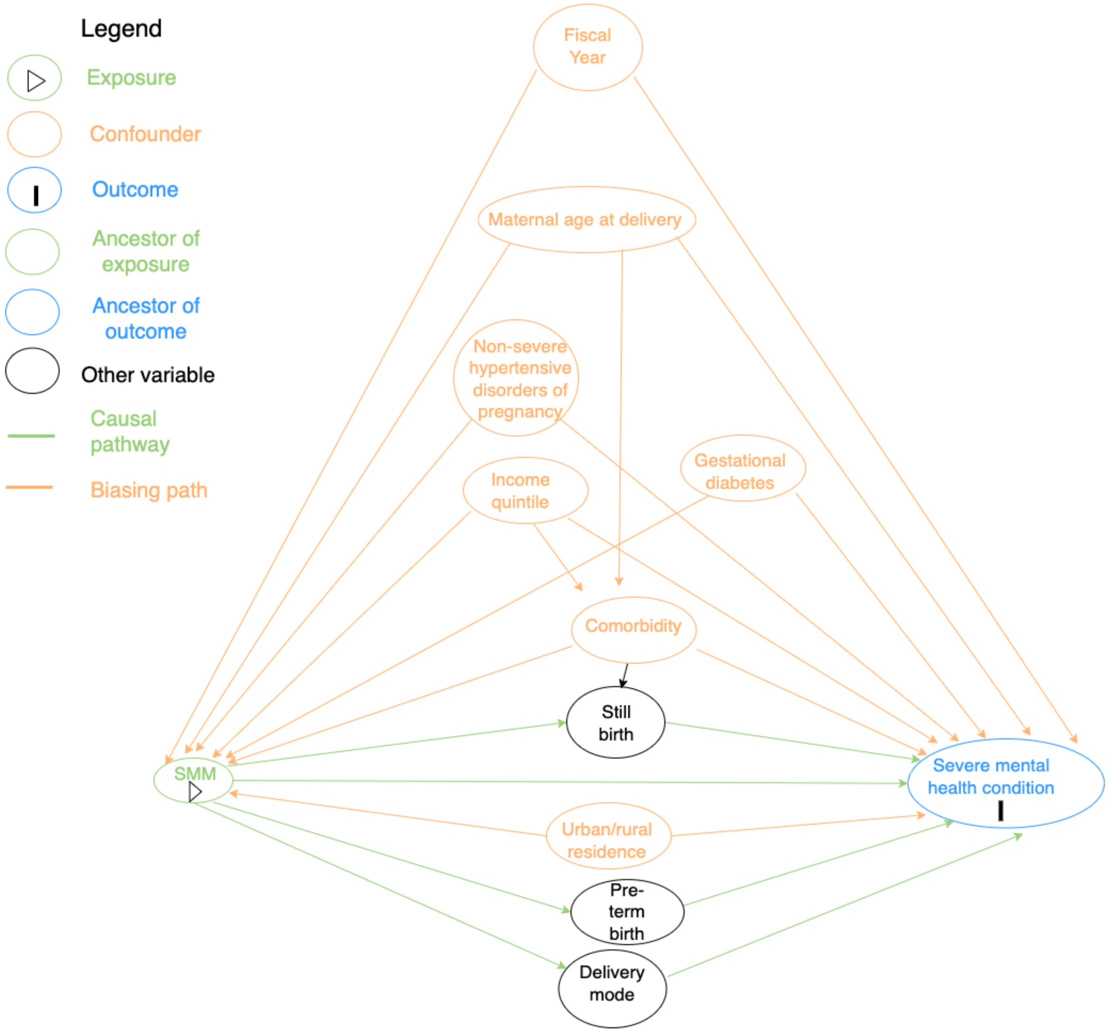

© 2024 Blackman A et al. *JAMA Network Open.*

eFigure 3. Frequency of severe maternal morbidity and rate per thousand births among hospital deliveries in Canada (excluding Quebec) during fiscal years 2006-07 through 2020-21, N = 4,232,185

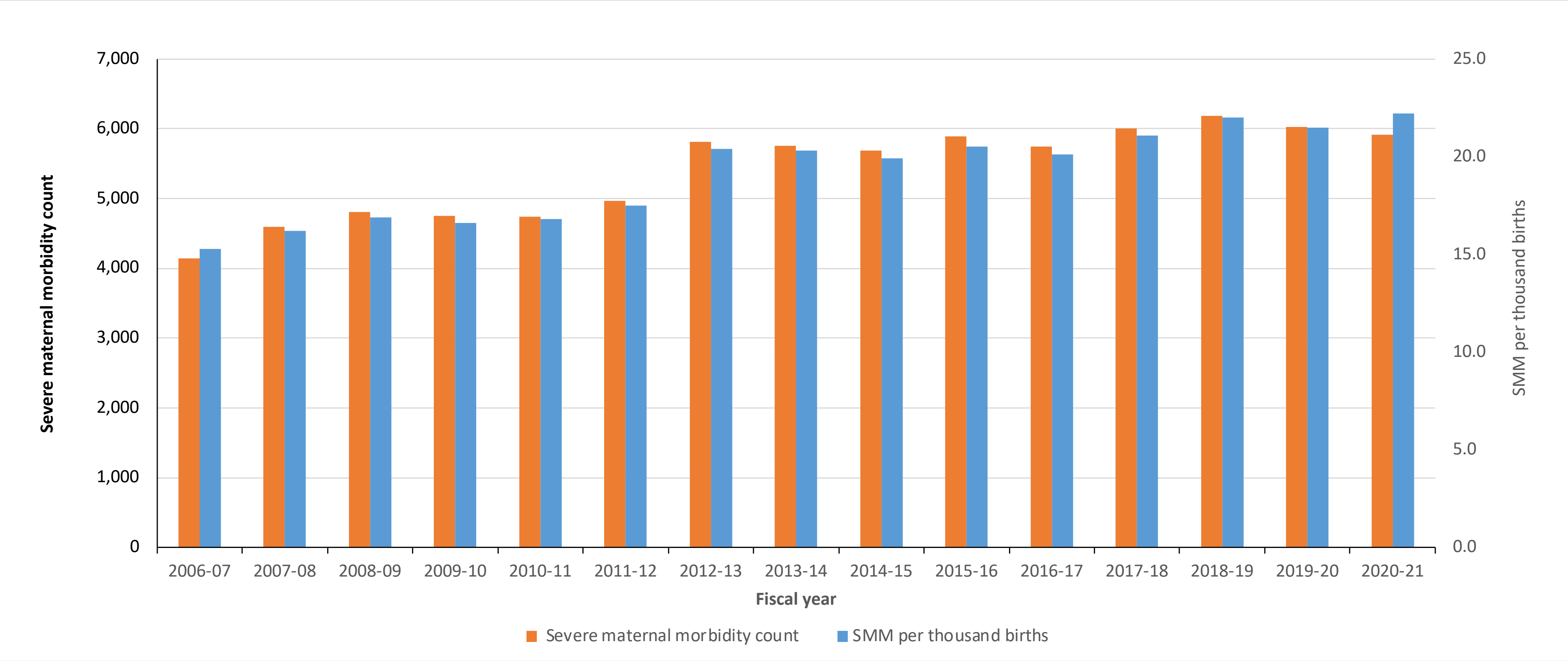

© 2024 Blackman A et al. *JAMA Network Open.*

eFigure 4. Forest plot of hazard ratios for the relationship between SMM and mental health hospitalization or ED visit, by province/territory

	Crude HR (95% CI)	*Adjusted HR (95% CI)*
Alberta (26.1 vs. 18.8, 352,340 births)	1.20 (1.07-1.37)	*1.16 (1.01-1.32)*
British Columbia (6.0 vs. 3.1, 323,383 births)	1.64 (1.21-2.24)	*Not available*
Manitoba (8.8 vs. 5.0, 102,206 births)	1.60 (1.03-2.47)	*Not available*
Nova Scotia (16.4 vs. 14.3, 61,890 births)	1.14 (0.75-1.74)	*Not available*
Ontario (42.9 vs. 31.5, 992,558 births)	1.33 (1.24-1.42)	*1.27 (1.19-1.36)*
Prince Edward Island (36.1 vs. 28.1, 9,296 births)	1.17 (0.37-3.69)	*Not available*
Saskatchewan (21.0 vs. 14.0, 93,100 births)	1.26 (0.94-1.70)	*1.22 (0.87-1.72)*
Territories (69.0 vs. 44.5, 9,116 births)	1.49 (0.92-2.42)	*Not available*
All except Ontario (15.9 vs. 10.7, 1,034,037 births)	1.33 (1.20-1.48)	*1.26 (1.14-1.39)*

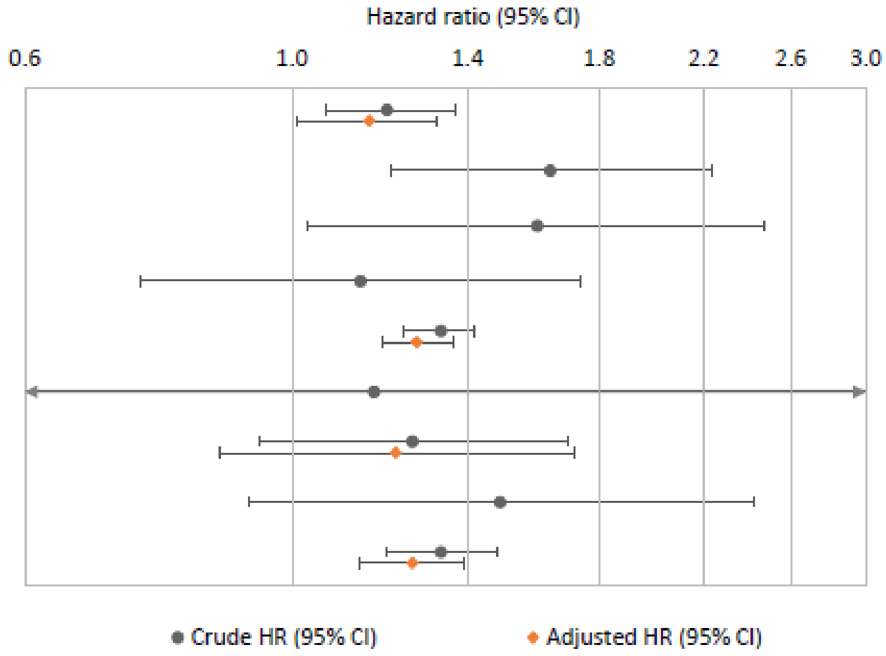

Note: Numbers in parentheses next to provinces represent rates of mental health hospitalization or ED visit per thousand deliveries with vs. without SMM, and total number of deliveries across the study period. Adjusted hazard ratios are shown for provinces where the number of women with both mental health hospitalization or ED visit and SMM is ≥45. Estimates were not reportable for New Brunswick and Newfoundland and Labrador due to small cell counts.

© 2024 Blackman A et al. *JAMA Network Open.*

eTable 1. Exposure definition with accompanying ICD-10 and CCI codes

SMM type	SMM subtype	ICD-10 CA and CCI codes
Severe preeclampsia, Hemolysis, Elevated liver enzymes and low platelets (HELLP) syndrome, eclampsia	Severe pre-eclampsia, HELLP syndrome	O14.1, O14.2
	Eclampsia	O15
Severe hemorrhage	Placenta previa with haemorrhage and red cell transfusion	O44.1 + RBCTRNSF='Y
	Antepartum hemorrhage with coagulation defect	O46.0
	Placental abruption with coagulation defect	O45.0
	Intrapartum hemorrhage with coagulation defect	O67.0
	Intrapartum hemorrhage with red cell transfusion	O67 + RBCTRNSF='Y'
	Postpartum hemorrhage with red cell transfusion,<br>procedures to the uterus or hysterectomy	O72 + any of the following:<br>RBCTRNSF='Y', or (1.RM.13, 1.KT.51, 5.PC.91.LA or 5.PC.91. HV) + RBCTRNSF = 1, or (5.MD.60.RC, 5.MD.60.RD, 5.MD.60.KE, 5.MD.60. CB or 1.RM.89.LAb), or 1.RM.87.LA-GX<br>Note: 1.RM.89.LA is included only if codes 1. PL.74, 1.RS.74 or 1.RS.80 are NOT also present
	Curettage with red cell transfusion	(5.PC.91.GA, 5.PC.91.GC or 5.PC.91. GD) + RBCTRNSF='Y
Maternal intensive care unit (ICU) admission	Maternal ICU admission	FTSPCU in ('10','20','25','30','35','40','45','60', '80')
Surgical complications	Complications of obstetric surgeries and procedures	O75.4
	Evacuation of incisional hematoma with RBC transfusion	5.PC.73.JS + RBCTRNSF='Y'
	Repair of bladder, urethra, or intestine	5.PC.80.JR, 1.NK.80 or 1.NM.80
	Reclosure of caesarean wound with RBC transfusion	(5.PC.80.JM or 5.PC.80.JH) + RBCTRNSF='Y'
Hysterectomy	Caesarean hysterectomy	5.MD.60.RC, 5.MD.60.RD, 5.MD.60.KE, 5.MD.60.CB
	Hysterectomy using an open approach (without bladder<br>neck suspension, suspension of vaginal vault or pelvic floor repair	1.RM.89.LAc (exclude if 1.PL.74, 1.RS.74 or 1.RS.80 code also present) or 1.RM.87.LA-GX<br>Note: 1.RM.89.LA is included only if codes 1.PL.74, 1. RS.74 or 1.RS.80 are NOT also present
Sepsis	Puerperal sepsis	O85
	Septicemia during labor	O75.3
Embolism, shock, disseminated intravascular coagulation (DIC)	Obstetric shock	O75.1, R57, T80.5 or T88.6
	Obstetric embolism	O88
	Disseminated intravascular coagulation	D65

© 2024 Blackman A et al. *JAMA Network Open.*

SMM type	SMM subtype	ICD-10 CA and CCI codes
Assisted ventilation	Assisted ventilation through endotracheal tube	1.GZ.31.CA-ND
	Assisted ventilation through tracheostomy	1.GZ.31.CR-ND
Cardiac conditions	Cardiac complications of anesthesia	O74.2, O89.1
	Cardiomyopathy	O90.3, I42, I43
	Cardiac arrest and resuscitation	I46, I49.0, 1.HZ.09, 1.HZ.30
	Myocardial infarction	I21, I22
	Pulmonary edema and heart failure	I50, J81
Acute renal failure	Acute renal failure	O90.4, N17, N19 or N99.0
	Dialysis	1.PZ.21
Severe uterine rupture	Rupture of the uterus with red cell transfusion,<br>procedures to the uterus or hysterectomy	(O71.0 or O71.1) + any of the following:<br>RBCTRNSF='Y', or<br>(1.RM.13, 1.KT.51, 5.PC.91.LA or 5.PC.91. HV) + RBCTRNSF='Y', or<br>(5.MD.60.RC, 5.MD.60.RD, 5.MD.60.KE, 5.MD.60. CB or 1.RM.89.LA), or<br>1.RM.87.LA-GX a<br><u>Note:</u> 1.RM.89.LA is included only if codes 1. PL.74, 1.RS.74 or 1.RS.80 are NOT also present
Cerebrovascular Accident	Cerebral venous thrombosis in pregnancy	O22.5
	Cerebral venous thrombosis in the puerperium	O87.3
	Subarachnoid and intracranial hemorrhage, cerebral infarction	I60, I61, I62, I63 or I64
Other types	Acute fatty liver with red cell transfusion or plasma transfusion	O26.6 + (RBCTRNSF='Y' or PLSTRNSF='Y')
	Hepatic failure	K71 or K72
	Cerebral edema or coma	G93.6 or R40.2
	Pulmonary, cardiac and CNS complications of anaesthesia during pregnancy, labour, delivery or the puerperium	O29.0, O29.1, O29.2, O89.0, O89.1, O89.2, O74.0, O74.1, O74.2 or O74.3
	Status asthmaticus	J45.01, J45.11, J45.81 or J45.91
	Adult respiratory distress syndrome	J80
	Acute abdomen	K35, K37, K65, N73.3 or N73.5
	Surgical or manual correction of inverted uterus for vaginal births only	5.PC.91.HQ or 5.PC.91.HP, restricted to vaginal births (i.e., absence of caesarean code 5.MD.60)
	Sickle cell anemia with crisis	D57.0
	Acute psychosis	F53.1 or F23
	Status epilepticus	G41
	HIV disease	B20-24, O98.7

© 2024 Blackman A et al. *JAMA Network Open.*

eTable 2. Outcome definition with accompanying ICD-10 and CCI codes

**Outcome**	**Individual diagnoses**	**ICD-10-CA codes**
Substance-Related and Addictive Disorders	Abuse of non-psychoactive substances	F55
	Abuse of psychoactive substances	F10 to F19
Mood and Anxiety Disorders	Mood disorder due to known physiological condition	F06.3
	Manic episode	F30
	Bipolar disorder	F31
	Depressive episode	F32
	Major depressive disorder, recurrent	F33
	Persistent mood (affective) disorders	F34
	Unspecified mood (affective) disorders	F39
	Anxiety disorder due to known physiological condition	F06.4
	Phobic anxiety disorders	F40
	Other anxiety disorders	F41
	Obsessive compulsive disorder	F42
	Reaction to severe stress, and adjustment disorders	F43
	Other specified nonpsychotic mental disorders	F48.8
	Nonpsychotic mental disorder, unspecified	F48.9
	Mental and behavioral disorders associated with the puerperium, not elsewhere classified	F53
Suicidality or Deliberate Self Harm	Intentional self-harm	X60-X84,
	Poisoning	Y10-Y19,
	Contact with sharp object	Y28,<br>Note when DX10CODE1 not equal<br>F06-F99 (DXTYPE = alldx or DXTYPE = 9)
Schizophrenia spectrum and other psychotic disorders	Psychotic disorder with hallucinations due to known physiological condition	F06.0
	Psychotic disorder with delusions due to known physiological condition	F06.2
	Schizophrenia	F20 (excl. F20.4)
	Delusional disorders	F22
	Brief psychotic disorder	F23
	Shared psychotic disorders	F24
	Schizoaffective disorders	F25
	Other psychotic disorder not due to substance or known physiological condition	F28

© 2024 Blackman A et al. *JAMA Network Open.*

Outcome	Individual diagnoses	ICD-10-CA codes
	Unspecified psychosis not due to a substance or known physiological condition	F29
	Puerperal psychosis	F53.1
Substance-related and addictive disorders	Alcohol related disorders	F10
	Opioid related disorders	F11
	Cannabis related disorders	F12
	Sedative, hypnotic or anxiolytic related disorders	F13
	Cocaine related disorders	F14
	Other stimulant related disorders	F15
	Hallucinogen related disorders	F16
	Nicotine dependence	F17
	Inhalant related disorders	F18
	Other psychoactive substance related disorders	F19
	Abuse of nonpsychoactive substances	F55

© 2024 Blackman A et al. *JAMA Network Open.*

eTable 3. Association between severe maternal morbidity and the risk of mental health hospitalization, or mental health ED visit, separately, by province or territory. N1=2,026,594 represents cohort examining hospitalizations and N2= 1,579,392 represents cohort examining ED visits.

	Mental Health Hospitalization$_1$					Mental Health ED visit$_2$				
Province	N	Total person-years	IRs per 10,000 person-years	Crude HR (95% CI)	Adjusted HR[a,b] (95% CI)	N	Total person-years	IRs per 10,000 person-years	Crude HR (95% CI)	Adjusted HR[a,b] (95% CI)
**Alberta**										
**Overall**	2663	1440683	18.5			6654	667651	99.7		
**Any SMM**	91	36763	24.8	1.35 (1.10-1.67)	1.28 (1.03-1.58)	227	19073	119.0	1.21 (1.07-1.38)	1.15 (1.01-1.32)
**No SMM**	2572	1403920	18.3	1.00 (Ref.)	Ref	6427	648578	99.1	1.00 (Ref.)	1.00 (Ref.)
**British Columbia**										
**Overall**	3181	1393596	22.8							
**Any SMM**	115	30152	38.1	1.67 (1.39-2.02)	1.68 (1.22-2.32)	≤5				
**No SMM**	3066	1363444	22.5	1.00 (Ref.)	Ref					
**Manitoba**										
**Overall**	950	409499	23.2							
**Any SMM**	31	9574	32.4	N/A	N/A	≤5				
**No SMM**	919	399925	23.0	N/A	N/A					
**New Brunswick**	31	9574	32.4							
**Overall**	512	221885	23.1							
**Any SMM**	20	5363	37.3	N/A	N/A	N/A	N/A	N/A	N/A	N/A
**No SMM**	492	216522	22.7	N/A	N/A	N/A	N/A	N/A	N/A	N/A
**Newfoundland and Labrador**										
**Overall**	260	163433	15.9							
**Any SMM**	8	4675	17.1	N/A	N/A	N/A	N/A	N/A	N/A	N/A
**No SMM**	252	158758	15.9	N/A	N/A	N/A	N/A	N/A	N/A	N/A
**Nova Scotia**										
**Overall**	455	283399	16.1			514	283389	18.14		
**Any SMM**	17	6108	27.8	N/A	N/A	7	6168	11.35	N/A	N/A
**No SMM**	438	277291	15.8	N/A	N/A	507	277221	18.29	N/A	N/A
**Ontario**										

© 2024 Blackman A et al. *JAMA Network Open.*

	Mental Health Hospitalization[1]					Mental Health ED visit[2]				
**Province**	**N**	**Total person-years**	**IRs per 10,000 person-years**	**Crude HR (95% CI)**	**Adjusted HR[a,b] (95% CI)**	**N**	**Total person-years**	**IRs per 10,000 person-years**	**Crude HR (95% CI)**	**Adjusted HR[a,b] (95% CI)**
**Overall**	1633	4233816	3.9			31503	4114876	76.6		
**Any SMM**	63	91480	6.9	1.81 (1.40-2.33)	1.68 (1.30-2.17)	892	88204	101.1	1.33 (1.24-1.42)	1.28 (1.19-1.36)
**No SMM**	1570	4142336	3.8	1.00 (Ref.)	Ref	30611	4026671	76.0	1.00 (Ref.)	Ref
**All provinces except Ontario**										
**Overall**	3800	1786850	21.3							
**Any SMM**	128	46579	27.5	1.33 (1.11-1.59)	1.28 (1.07-1.54)	N/A	N/A	N/A	N/A	N/A
**No SMM**	3672	1740272	21.1	1.00 (Ref.)	Ref	N/A	N/A	N/A	N/A	N/A
**Prince Edward Island**										
**Overall**										
**Any SMM**	≤5					≤5				
**No SMM**										
**Saskatchewan**										
**Overall**	1189	371291	32.0			1145	141157	81.1		
**Any SMM**	35	8556	40.9	N/A	N/A	42	3610	116.3	N/A	N/A
**No SMM**	1154	362734	31.8	N/A	N/A	1103	137547	80.2	N/A	N/A
**Territories**										
**Overall**						261	40119	65.1		
**Any SMM**	≤5					14	1124	124.6	N/A	N/A
**No SMM**						247	38995	63.3	N/A	N/A

[a]Hazard ratios adjusted for maternal age at delivery, income quintile, comorbidity, delivery year and urban or rural residential status

[b]Adjusted hazard ratios are shown for provinces where the number of women with both mental health hospitalization or ED visit and SMM is ≥45.

Abbreviations: IR - incidence rate, ED - emergency department, HR - hazard ratio, SMM - severe maternal morbidity

© 2024 Blackman A et al. *JAMA Network Open.*

eTable 4. Association between severe maternal morbidity and the risk of hospitalization and/or ED visit capturing a mental health condition recorded in any of the twenty-five diagnostic fields during hospitalization, N=1,579,392

SMM	Total mental health hospitalization or ED visits (n)	Total person-years	Incidence rate per 10,000 person-years	Crude HR (95% CI)	Adjusted HR[a] (95% CI)
**Any**	1479	133200	111.0	1.39 (1.32-1.46)	1.32 (1.26-1.39)
**None**	45532	5732934	79.4	1.00 (Ref.)	1.00 (Ref.)

[a] Hazard ratios adjusted for maternal age at delivery, income quintile, comorbidity, delivery year and urban or rural residential status

Abbreviations: ED - emergency department, HR - hazard ratio, SMM - severe maternal morbidity

© 2024 Blackman A et al. *JAMA Network Open.*

eTable 5. Association between severe maternal morbidity and the risk of mental health hospitalization or ED visit among individuals with previous mental illness, N=38,771

Characteristic	Total mental health hospitalization or ED visits (n)	Total person-years	Incidence rate per 10,000 person-years	Crude HR (95% CI)	Adjusted HR[a] (95% CI)
**Overall**	10149	126949	799.5		
**Any SMM**	429	4041	1061.6	1.30 (1.18-1.44)	1.21 (1.10-1.34)
**No SMM**	9720	122908	790.8	1.00 (Ref.)	1.00 (Ref.)

[a] Hazard ratios adjusted for maternal age at delivery, income quintile, comorbidity, delivery year and urban or rural residential status

Abbreviations: ED - emergency department, HR - hazard ratio, SMM - severe maternal morbidity

© 2024 Blackman A et al. *JAMA Network Open.*

eTable 6. Association between severe maternal morbidity and the risk of mental health hospitalization or ED visit without censoring on subsequent pregnancy, N=1,579,392

Characteristic	Total mental health hospitalization or ED visits (n)	Total person-years	Incidence rate per 10,000 person-years	Crude HR (95% CI)	Adjusted HR[a] (95% CI)
**Overall**	67169	9226945	72.8		
**Any SMM**	1874	193992	96.6	1.33 (1.27-1.39)	1.27 (1.21-1.33)
**No SMM**	65295	9032953	72.3	1.00 (Ref.)	1.00 (Ref.)

[a] Hazard ratios adjusted for maternal age at delivery, income quintile, comorbidity, delivery year and urban or rural residential status

Abbreviations: ED - emergency department, HR - hazard ratio, SMM - severe maternal morbidity

© 2024 Blackman A et al. *JAMA Network Open.*

eTable 7. Association between severe maternal morbidity and the risk of mental health hospitalization or ED visit in a cohort where stillbirth and preterm birth are excluded, N= 1,449,893

Characteristic	Total mental health hospitalization or ED visits (n)	Total person-years	Incidence rate per 10,000 person-years	Crude HR (95% CI)	Adjusted HR[a] (95% CI)
**Overall**	38448	5373825	71.6		
**Any SMM**	926	99056	93.5	1.30 (1.22-1.39)	1.26 (1.18-1.34)
**No SMM**	37522	5274768	71.1	1.00 (Ref.)	1.00 (Ref.)

[a] Hazard ratios adjusted for maternal age at delivery, income quintile, comorbidity, delivery year and urban or rural residential status

Abbreviations: ED - emergency department, HR - hazard ratio, SMM - severe maternal morbidity

© 2024 Blackman A et al. *JAMA Network Open.*

eTable 8. Association between severe maternal morbidity at or before delivery and the risk of mental health hospitalization or ED visit.

Characteristic	Total mental health hospitalization or ED visits (n)	Total person-years	Incidence rate per 10,000 person-years	Crude HR (95% CI)	Adjusted HR[a] (95% CI)
**Overall**	45359	6051960	71.6		
**Any SMM**	1043	109323	93.5	1.27 (1.20-1.36)	1.22 (1.14-1.30)
**No SMM**	44316	5942637	71.1	1.00 (Ref.)	1.00 (Ref.)

[a] Hazard ratios adjusted for maternal age at delivery, income quintile, comorbidity, delivery year and urban or rural residential status

Abbreviations: ED - emergency department, HR - hazard ratio, SMM - severe maternal morbidity

© 2024 Blackman A et al. *JAMA Network Open.*

eTable 9. Association between severe maternal morbidity and the risk of mental health-related hospitalization or ED visit within the first year postpartum, from 1 year to 5 years postpartum, and beyond 5 years postpartum, N=1,579,392

Follow-up Time	Total mental health hospitalization or ED visits (n)	Total person-years	Incidence rate per 10,000 person-years	Crude HR (95% CI)	Adjusted HR[a] (95% CI)
**≤1 year**					
**Overall**	10885	173254	628.3		
**Any SMM**	356	3707	960.3	1.46 (1.31-1.62)	1.38 (1.24-1.53)
**No SMM**	10529	169547	621.0	1.00 (Ref.)	1.00 (Ref.)
**>1 year and ≤5 years**					
**Overall**	22491	2057417	109.3		
**Any SMM**	670	47458	141.2	1.27 (1.18-1.38)	1.23 (1.14-1.34)
**No SMM**	21821	2009960	108.6	Ref	1.00 (Ref.)
**>5 years**					
**Overall**	9690	3649064	26.6		
**Any SMM**	261	82804	31.5	1.25 (1.10-1.41)	1.21 (1.07-1.37)
**No SMM**	9429	3566250	26.4	1.00 (Ref.)	1.00 (Ref.)

[a] Hazard ratios adjusted for maternal age at delivery, income quintile, comorbidity, delivery year and urban or rural residential status

Abbreviations: ED - emergency department, HR - hazard ratio, SMM - severe maternal morbidity

© 2024 Blackman A et al. *JAMA Network Open.*

eTable 10. Baseline characteristics of study cohort at first recorded hospital delivery, presented according to those with complete and incomplete (missing) data.[a,b]

Characteristic	Complete (N = 1541105)	Incomplete (N = 38287)	Standardized difference
**Maternal age at delivery (y)**			
**Mean (SD)**	30.0 (5.4)	29.6 (5.6)	
**18-24**	249108 (16.2)	7395 (19.3)	0.08
**25-29**	464005 (30.1)	11116 (29.0)	0.02
**30-34**	519085 (33.7)	12459 (32.5)	0.03
**35-39**	247831 (16.1)	5867 (15.3)	0.02
**40-44**	56580 (3.7)	1352 (3.5)	0.01
**≥45**	4496 (0.3)	98 (0.3)	0.00
**Income quintile**			
**Lowest quintile**	361978 (23.5)	606 (27.4)	0.09
**Second quintile**	323510 (21.0)	477 (21.6)	0.01
**Third quintile**	320807 (20.8)	409 (18.5)	0.06
**Fourth quintile**	301023 (19.5)	402 (18.2)	0.03
**Highest quintile**	233787 (15.2)	313 (14.2)	0.03
**Missing**	0	36080	N/A
**Delivery Year**			
**2008-09**	114303 (7.4)	2800 (7.3)	0.00
**2009-10**	97923 (6.4)	2462 (6.4)	0.00
**2010-11**	86841 (5.6)	2117 (5.5)	0.00
**2011-12**	83936 (5.4)	1636 (4.3)	0.05
**2012-13**	114521 (7.4)	1887 (4.9)	0.10
**2013-14**	117978 (7.7)	2223 (5.8)	0.08
**2014-15**	140719 (9.1)	2673 (7.0)	0.08
**2015-16**	138733 (9.0)	2427 (6.3)	0.10
**2016-17**	136873 (8.9)	2613 (6.8)	0.08
**2017-18**	134002 (8.7)	3435 (9.0)	0.01
**2018-19**	131133 (8.5)	4216 (11.0)	0.08

Characteristic	Complete<br>(N = 1541105)	Incomplete<br>(N = 38287)	Standardized difference
**2019-20**	132351 (8.6)	5341 (13.9)	0.17
**2020-21**	111792 (7.3)	4457 (11.6)	0.15
**Province**			
**Alberta**	637612 (21.1)	12240 (24.6)	0.08
**British Colombia**	150048 (5.0)	2120 (4.3)	0.03
**Manitoba**	203384 (6.7)	3885 (7.8)	0.04
**New Brunswick**	27161 (0.9)	437 (0.9)	0.00
**Newfoundland and Labrador**	16267 (0.5)	277(0.6)	0.01
**Nova Scotia**	33451(1.1)	547 (1.1)	0.00
**Ontario**	1738856 (57.7)	26384 (52.9)	0.10
**Prince Edward Island**	8931 (0.3)	134 (0.3)	0.00
**Saskatchewan**	182168 (6.0)	3376 (6.8)	0.03
**Northern Territories**	18104 (0.6)	443 (0.9)	0.03
**Urban/rural residence**			
**Urban**	1368266 (88.8)	2337 (79.6)	0.05
**Rural/Remote**	172839 (11.2)	598 (20.4)	0.05
**Missing**	0	35352	N/A
**Hospital type**			
**Teaching tertiary hospital**	636080 (41.3)	1150 (42.1)	0.02
**Community**	905025 (58.7)	15866 (57.9)	0.02
**Missing**	0	10871	N/A
**Comorbidity**			
**0**	1323631 (85.9)	33500 (87.5)	0.05
**1**	197834 (12.8)	4468 (11.7)	0.03
**≥2**	19640 (1.3)	319 (0.8)	0.05
**Non-severe hypertensive disorders of pregnancy**			
**Yes**	91375 (5.9)	1900 (5.0)	0.04
**No**	1449730 (94.1)	36387 (95.0)	0.04
**Gestational diabetes**			

© 2024 Blackman A et al. *JAMA Network Open.*

Characteristic	Complete (N = 1541105)	Incomplete (N = 38287)	Standardized difference
**Yes**	128428 (8.3)	2952 (7.7)	0.02
**No**	1412677 (91.7)	35335 (92.3)	0.02
**Gestational age at delivery (weeks)**			
**Median (IQR)**	39 (38-40)	38 (38-40)	
**≤22**	3544 (0.2)	92 (0.2)	0.00
**22-27**	10494 (0.7)	311 (0.8)	0.01
**28-32**	15486 (1.0)	344 (0.9)	0.01
**33-36**	94716 (6.1)	2303 (6.1)	0.00
**≥37**	1416865 (91.9)	34742 (91.9)	0.00
**Missing**	0	495	N/A
**Stillbirth**			
**Yes**	7800 (0.5)	252 (0.7)	0.03
**No**	1533225 (99.5)	38035 (99.3)	0.03
**Delivery mode**			
**Cesearean**	486012 (31.5)	11198 (29.2)	0.05
**Obstetric delivery not otherwise specified**	214025 (13.9)	4647 (12.1)	0.05
**Vaginal**	841068 (54.6)	22442(58.6)	0.08
**Severe maternal morbidity**			
**Any SMM**	34979 (2.3)	846 (2.2)	0.01
**No SMM**	1506126(97.7)	37441(97.8)	0.01

[a]Data are presented as N (%) unless otherwise specified.

[b]Missing values were excluded when calculating percentages

© 2024 Blackman A et al. *JAMA Network Open.*
