# Supplementary material for: Severe Maternal Morbidity and Mental Health Hospitalizations or Emergency Department Visits
Source: JAMA Netw Open. 2024 Apr 23;7(4):e247983. doi: 10.1001/jamanetworkopen.2024.7983 (PMC11040413; doi:10.1001/jamanetworkopen.2024.7983)
Supplement: Supplement 2. — Data Sharing Statement [file jamanetwopen-e247983-s002.pdf]

Data Sharing Statement

Blackman. Severe Maternal Morbidity and Mental Health Hospitalizations or Emergency Department Visits. *JAMA Netw Open*. Published April 23, 2024.
doi:10.1001/jamanetworkopen.2024.7983

Data
Data available: No

Additional Information
Explanation for why data not available: Due to privacy and security restrictions for Canadian hospitalization data (Canadian Institute for Health Information), we are unable to offer access to individual patient data.
